# Electronic structures of WS_2_ armchair nanoribbons doped with transition metals

**DOI:** 10.1038/s41598-020-73602-2

**Published:** 2020-10-05

**Authors:** Yan-Hong Chen, Chi-Hsuan Lee, Shun-Jen Cheng, Chih-Kai Yang

**Affiliations:** 1 Department of Electrophysics, National Chiao Tung University, Hsinchu, Taiwan, ROC; 2 Graduate Institute of Applied Physics, National Chengchi University, Taipei, Taiwan, ROC

**Keywords:** Materials science, Physics

## Abstract

Armchair WS_2_ nanoribbons are semiconductors with band gaps close to 0.5 eV. If some of the W atoms in the ribbon are replaced by transition metals, the impurity states can tremendously affect the overall electronic structure of the doped ribbon. By using first-principles calculations based on density functional theory, we investigated substitutional doping of Ti, V, Cr, Mn, Fe, and Co at various positions on WS_2_ ribbons of different widths. We found that Fe-doped ribbons can have two-channel conduction in the middle segment of the ribbon and at the edges, carrying opposite spins separately. Many Co-doped ribbons are transformed into spin filters that exhibit 100% spin-polarized conduction. These results will be useful for spintronics and nanoelectronic circuit design.

Transition-metal dichalcogenides (TMDCs)^1^ have layered crystalline structures and a variety of chemical compositions. The relatively weak van der Waals interactions between adjacent layers facilitate extraction and synthesis of TMDC monolayers^2–10^, which have direct band gaps and remarkable electrical and optical properties^11–16^. TMDC monolayers can be tailored to nanoribbons^9,10^ that have atoms of either species at the two edges. The contribution of the edge atoms to the overall electronic structure and associated physical properties can be more important than the contribution from bulk atoms, those well inside the nanoribbon.

WS_2_ nanoribbons can be synthesized with controllable thickness by, for example, conversion^17^ of a pre-patterned H-terminated Si layer to metallic W by WF_6_, followed by in situ sulfidation by H_2_S. Using single- and double-walled carbon nanotubes as templates, ultra-narrow WS_2_ armchair-edged and zigzag-edged nanoribbons^18^ can also be produced, with a width as thin as 1–3 nm. Metallic WS_2_ nanoribbons with ammonia-ion intercalation can function as highly conductive electrodes^19^ for high-performance supercapacitors. Theoretical calculations based on density functional theory (DFT) have shown that zigzag WS_2_ nanoribbons exhibit ferromagnetic–metallic behavior, whereas armchair nanoribbons are semiconductors^20–22^, in good agreement with experiments. DFT calculations have also predicted that WS_2_ and MoS_2_ nanoribbons can form in-plane heterostructures^23,24^ with rectifying and/or spin-filtering functions^24^, rendering them attractive nanoelectronic devices. A theoretical study^25^ obtained a very large thermal magnetoresistance value and excellent spin filtration for a WS_2_ nanoribbon sandwiched between two electrodes that featured a temperature differential.

We present an investigation of WS_2_ nanoribbons with armchair edges, which are semiconductors with sizable band gaps. Doping by impurities^26–31^, as is commonly practiced with semiconductors, can greatly alter the electronic structure. We are particularly interested in substitutional doping by transitional metals (TMs), where a W atom is replaced by a TM, and it is thus endowed with a *d* shell of electrons and many means for effecting a change in physical properties. Experimental studies show that Cr and Fe substitutional dopants, for example, greatly influence the electronic and optical properties of WS_2_ flakes^31^.

## Results and discussion

### Intrinsic armchair WS_2_ nanoribbons

**Figure 1:**
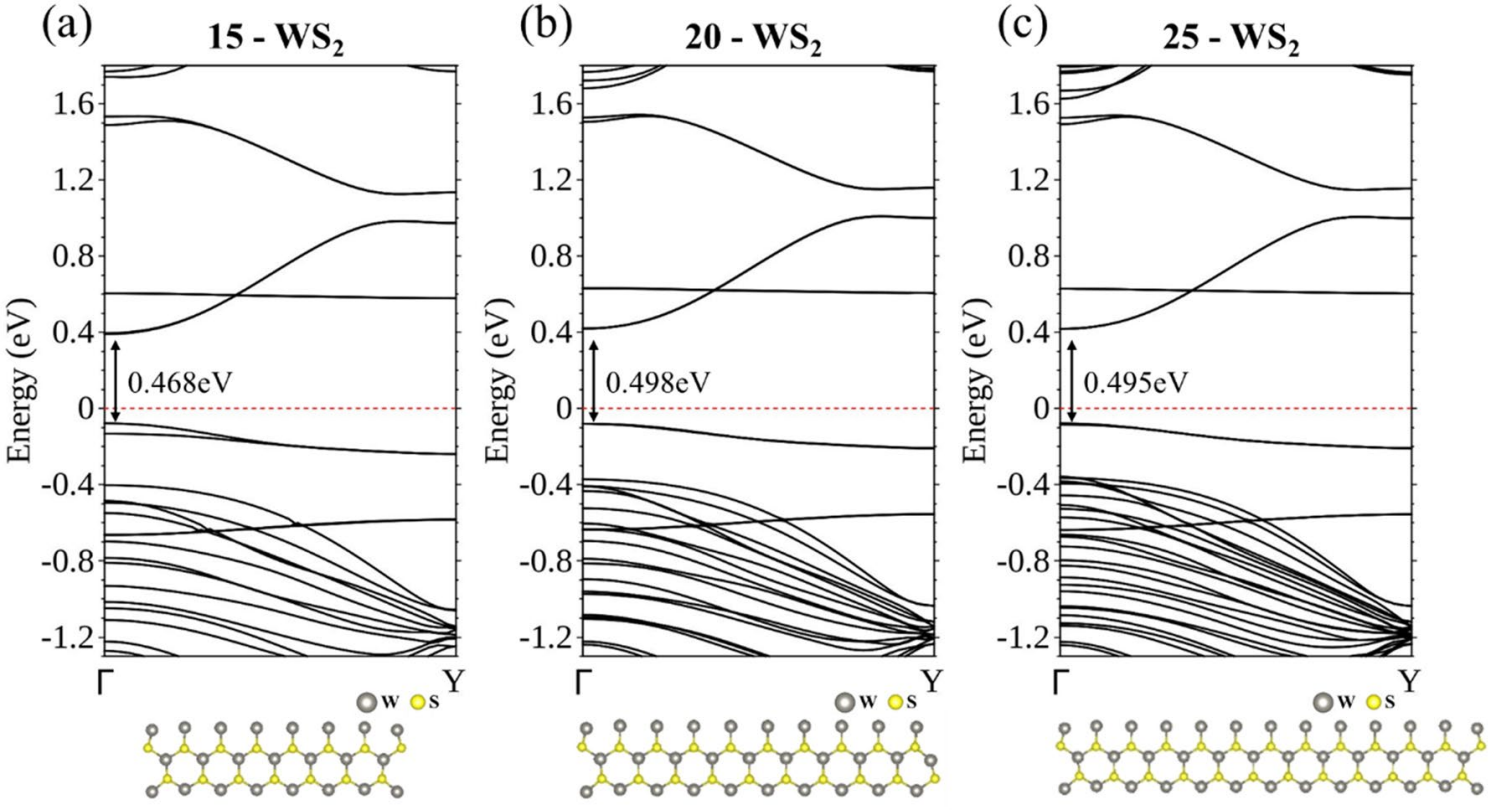
Energy bands of *N*a-WS_2_ nanoribbons, where *N*a is (**a**) 15, (**b**) 20, and (**c**) 25.

We first present energy bands of intrinsic *N*a-WS_2_ nanoribbons, where *N*a is 15, 20, or 25, representing ribbons of three different widths. Each WS_2_ ribbon [*N*a = 15, 20, and 25 in Fig. 1a–c, respectively] manifests its finite width by valence and conduction bands mainly associated with W atoms at its armchair edges, including a flat band (Fig. 1a–c) at approximately 0.6 eV. The contribution from the S atoms at the edges also forms a nearly flat band that is much lower in energy and mainly within the energy bands of the bulk. The band gap is 0.468 eV for 15-WS_2_, increases to 0.498 eV for 20-WS_2_, and then stays almost unchanged for the even wider 25-WS_2_.

### Doping at the center of 15-WS_2_

**Figure 2:**
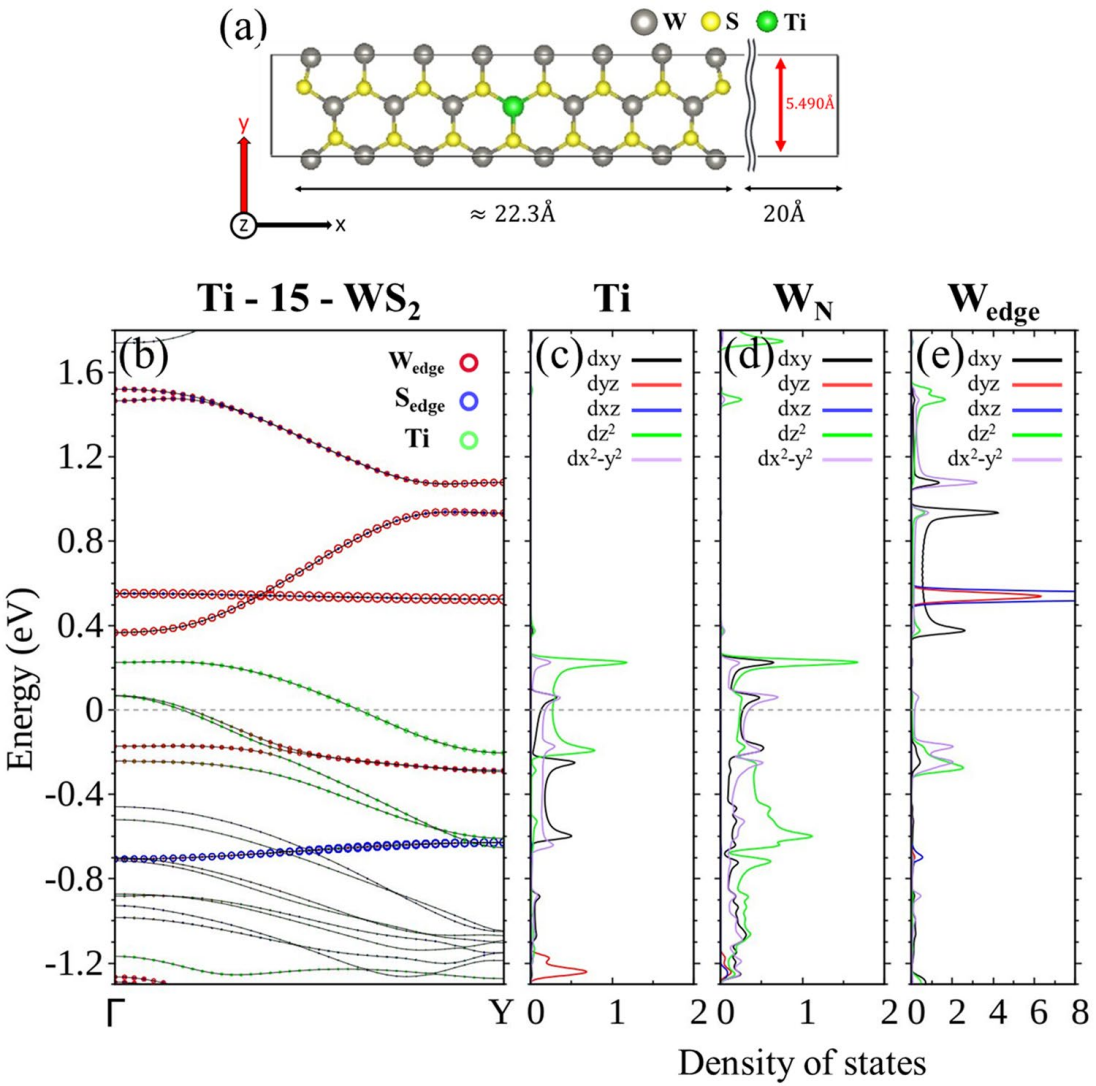
(**a**) Unit cell and (**b**) energy bands of Ti-15-WS_2_. LDOS in partial waves of (**c**) Ti, (**d**) W atoms closest to Ti, and (**e**) W at the edges.

We next discuss the configuration in which the substitutional doping occurs in the central position of the 15-WS_2_ ribbon and a W atom is replaced by a Ti atom at a concentration of 1/15 (6.67%). Figure 2 plots the exact position of the substitution and calculated electronic structure. Compared with the pristine 15-WS_2_, the most striking features are three (with two almost degenerate) energy bands crossing the Fermi level *E*_F_, rendering the centrally doped Ti-15-WS_2_ structure a conductor. The band with the highest energy of the three is almost exclusively composed of the *d* orbitals from the Ti impurity and its nearest neighbors of W atoms, which are marked collectively by green spheres in Fig. 2b with sizes proportional to their contributions, and also curves of local density of states (LDOS) in partial waves in Fig. 2c,d. The other two bands are likewise derived heavily from the *d* orbitals of the Ti, but a conspicuous presence of edge W atoms (as indicated by brown spheres in Fig. 2b and the corresponding LDOS in Fig. 2e) also emerges, largely below *E*_F_.

We therefore established that conduction of the centrally doped Ti-15-WS_2_ ribbon at a concentration of 6.67% is overwhelmingly carried out by the Ti and its neighboring W atoms, whereas the basic architecture of the pure WS_2_ ribbon energy bands consisting of edge W and S atoms remains largely intact. The wave function of Ti does not have an appreciable overlapping with those of W atoms at both edges. Analysis of the *d* orbitals by partial waves further identifies the three major constituents in *d*_xy_, *d*_x2−y2_, and *d*_z2_.

**Figure 3:**
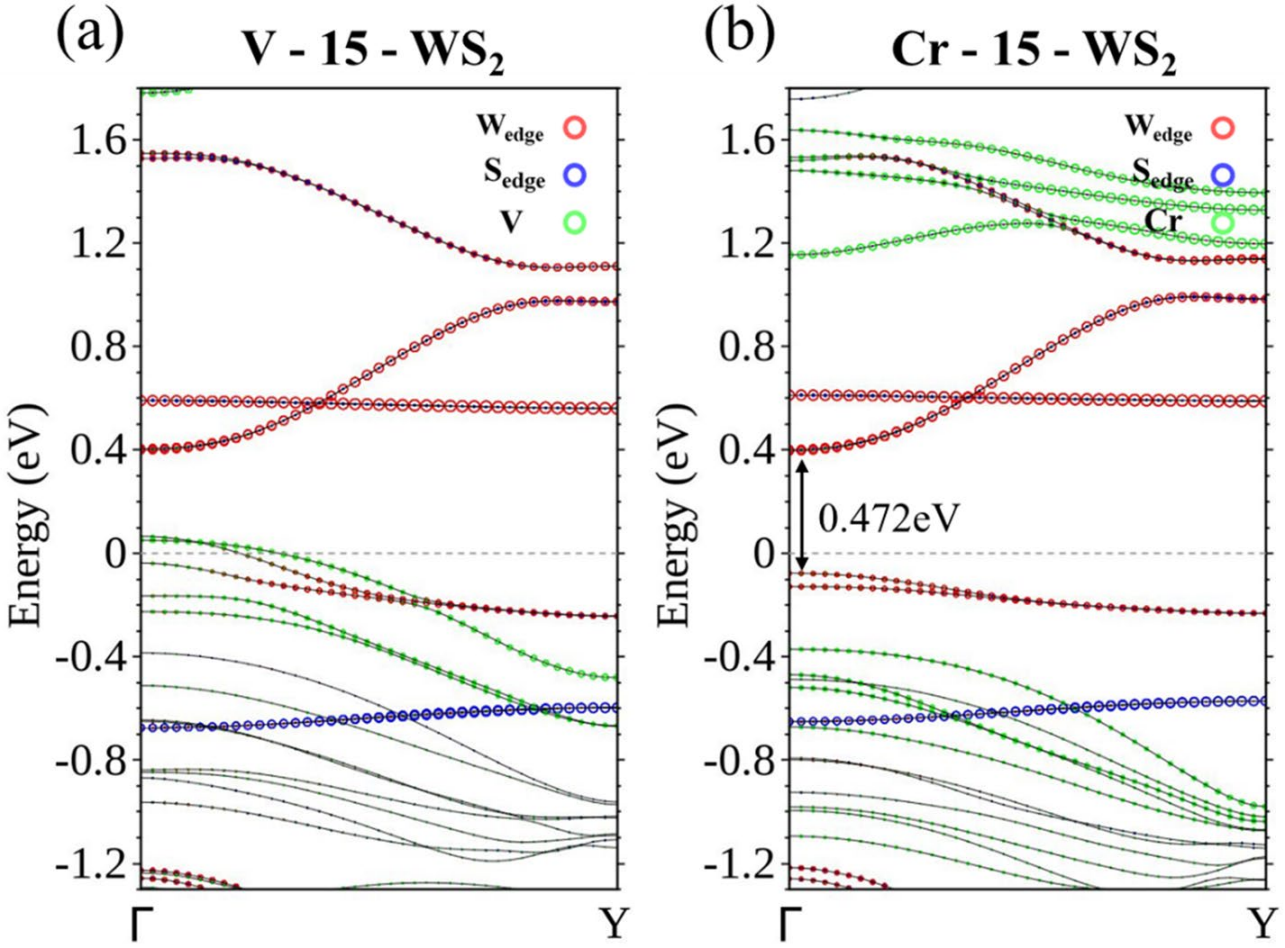
Energy bands of centrally doped (**a**) V-15-WS_2_ and (**b**) Cr-15-WS_2_.

Many of the descriptions of the centrally doped Ti-15-WS_2_ can also be applied to V-15-WS_2_. The dopant V, however, has one more electron in its *d* shells, or one hole less as compared with Ti. Implied in the rigid band estimate is that fewer bands would cross *E*_F_ as a result. This is exactly what the density functional calculation shows in Fig. 3a, in which only two bands cross *E*_F_ and most of their electronic states are below *E*_F_. The reduction in the available states also proceeds through the impurity and its surrounding W atoms, where the wave functions have the same *d* components just as those in the Ti case.

Having exactly the same electrons in the *d* shell as W, Cr as a dopant is not expected to alter the electrical property of the pure WS_2_ ribbon. The bands contributed by Cr either stay well below *E*_F_ or are pushed high above *E*_F_, with little mixing between the orbitals of Cr and W atoms at the edges (Fig. 3b). Centrally doped Cr-15-WS_2_ remains a semiconductor.

**Figure 4:**
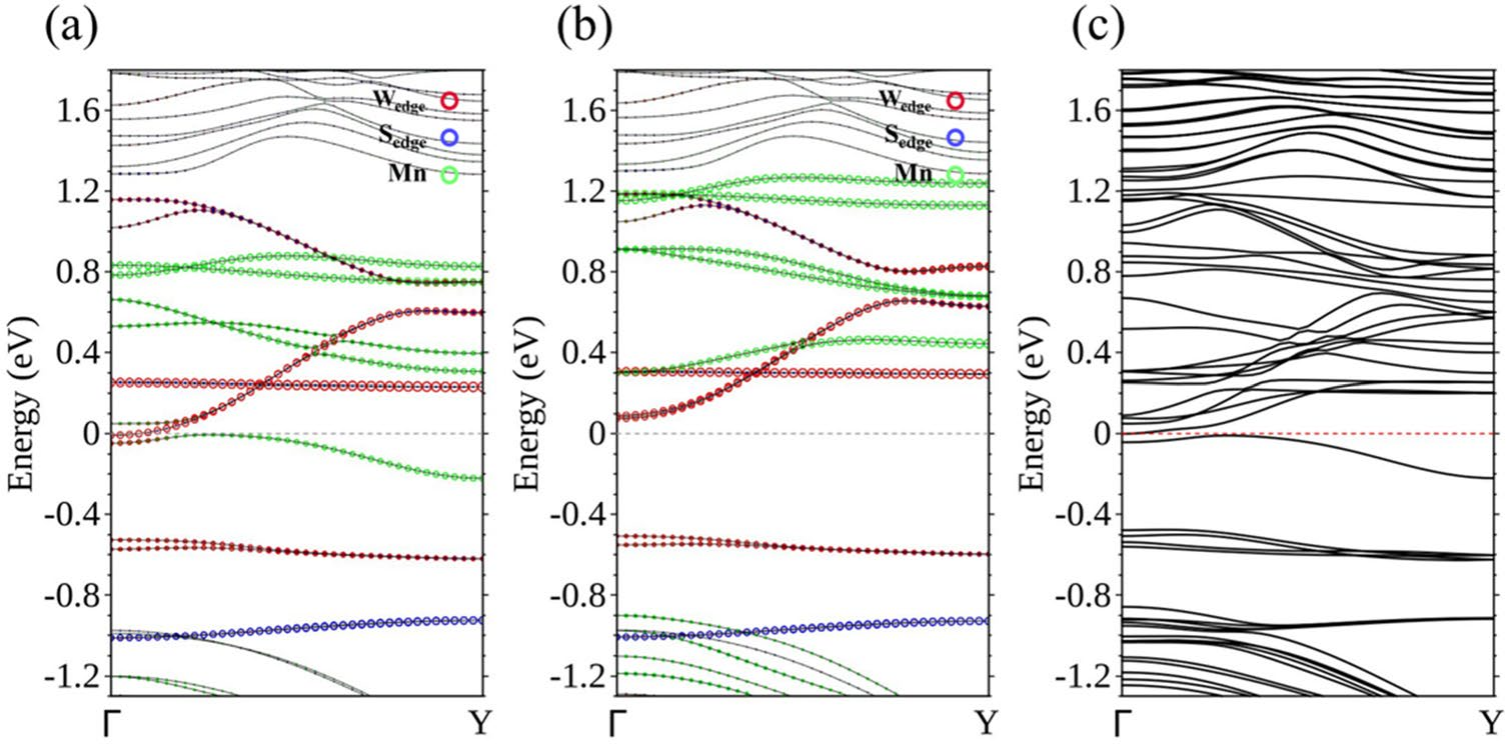
Energy bands of Mn-15-WS_2_ for the (**a**) majority spin and (**b**) minority spin. (**c**) Energy bands of Mn-15-WS_2_ with spin–orbit interaction.

Ti, V, and Cr-doped WS_2_ ribbons are essentially non-magnetic, but doping with Mn, Fe, and Co, classified as N-type, renders the ribbon magnetic. Using spin-polarized calculation, we are able to separate the energy bands corresponding to the majority spin (Fig. 4a) from those of the minority spin (Fig. 4b), for the 15-WS_2_ doped with a Mn in the center. Figure 4a clearly identifies two bands associated with the majority spin approaching *E*_F_ from above and below separately. The former is contributed by edge W atoms and the latter mainly by the Mn and its neighboring W atoms. The two curves are flat when they are closest to the Fermi level and it is likely that these states will not contribute to conduction in an electronic transport setup. No bands of the minority spin come so close to *E*_F_ in Fig. 4b. The imbalance of the distribution of spins produces a magnetic moment of 0.938 *μ*_*B*_ per unit cell for the doped ribbon. To further clarify the band structure near *E*_F_, we also undertook calculations that incorporated the spin–orbit interaction. The result supports the idea of a semiconductor with an indirect band gap of approximately 60 meV (Fig. 4c).

**Figure 5:**
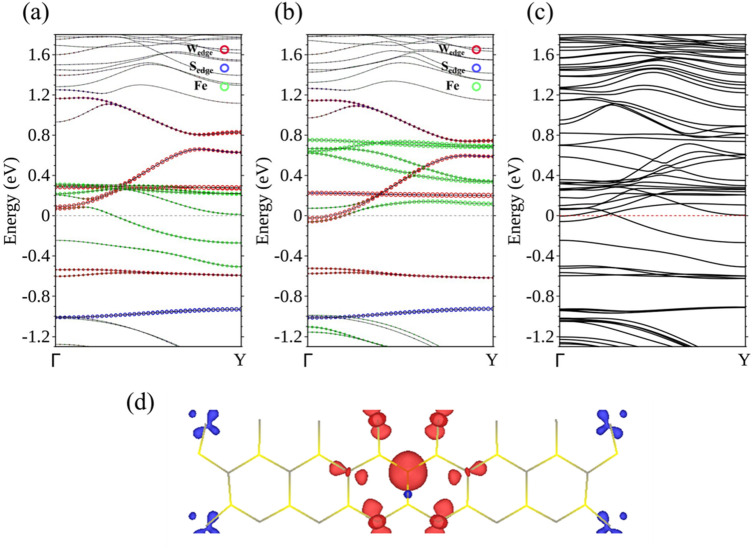
Energy bands of Fe-15-WS_2_ for the (**a**) majority spin and (**b**) minority spin. (**c**) Energy bands of Fe-15-WS_2_ with spin–orbit interaction. (**d**) Spin density distribution of Fe-15-WS_2_, with red representing the majority spin and blue the minority spin.

Proceeding next to Fe doping, which has one more electron available per unit cell, the electronic structure is very different from the previous examples. The 15-WS_2_ with a centrally doped Fe is now a conductor with two bands crossing *E*_F_. Each band is associated with the conductance of one quantum unit *e*^2^/*h* in a ballistic regime for a perfect periodic system, where *e* is the electron charge and *h* is Planck constant. The band with the majority spin (Fig. 5a) is comprised of the orbitals mainly from Fe and its surrounding W, indicating available states in the central region of the ribbon. The band of the minority spin (Fig. 5b) is strongly associated with W atoms at both edges. Mixing of wave functions between Fe and the edges generally occurs at energies greater than *E*_F_. Thus, even though charge transport can occur both in the interior and edges of the ribbon, the associated spins are separately carried by electrons in the two channels, with the former bearing the majority spin and the latter the minority spin. The entire unit cell of the ribbon exhibits a magnetic moment of 1.128 *μ*_*B*_. A further calculation taking into account the spin–orbit interaction also confirms this two-channel transport scenario (Fig. 5c). Figure 5d shows the distribution of spin density, where the majority spin occupying the central region of the ribbon is clearly separated from the minority spin confined at the edges.

**Figure 6:**
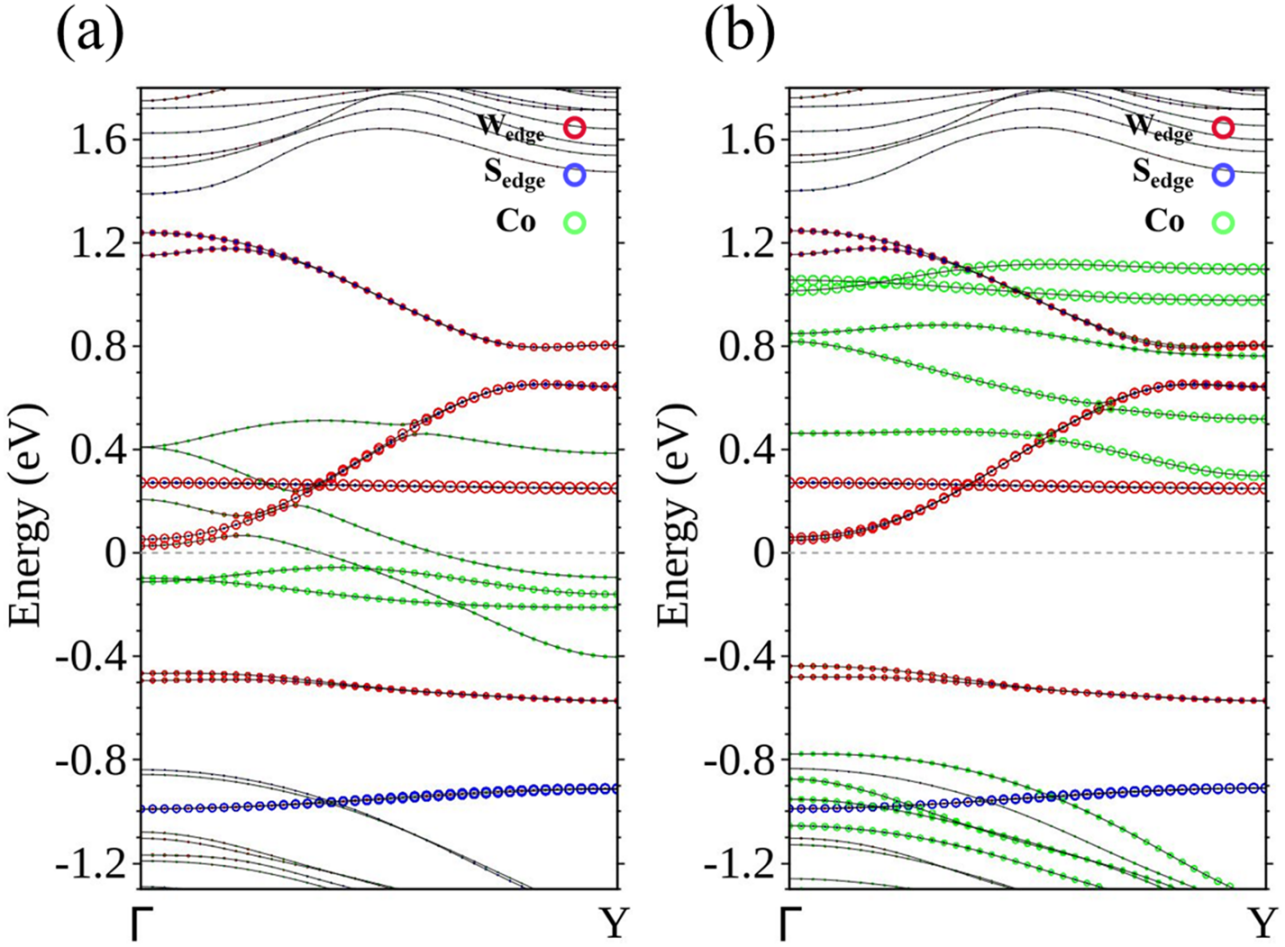
Energy bands of Co-15-WS_2_ for the (**a**) majority spin and (**b**) minority spin.

Continuing the study of TM-doped 15-WS_2_ nanoribbons, we now substitute a Co for the W atom at the central position of the unit cell. The calculated band structure is surprising in terms of its physical significance. There are now two bands crossing *E*_F_, both exclusively associated with the majority spin (Fig. 6a), but there are none doing so for the minority spin (Fig. 6b). The ribbon is therefore transformed into a spin filter, allowing only passage of electrons that have the majority spin.

**Figure 7:**
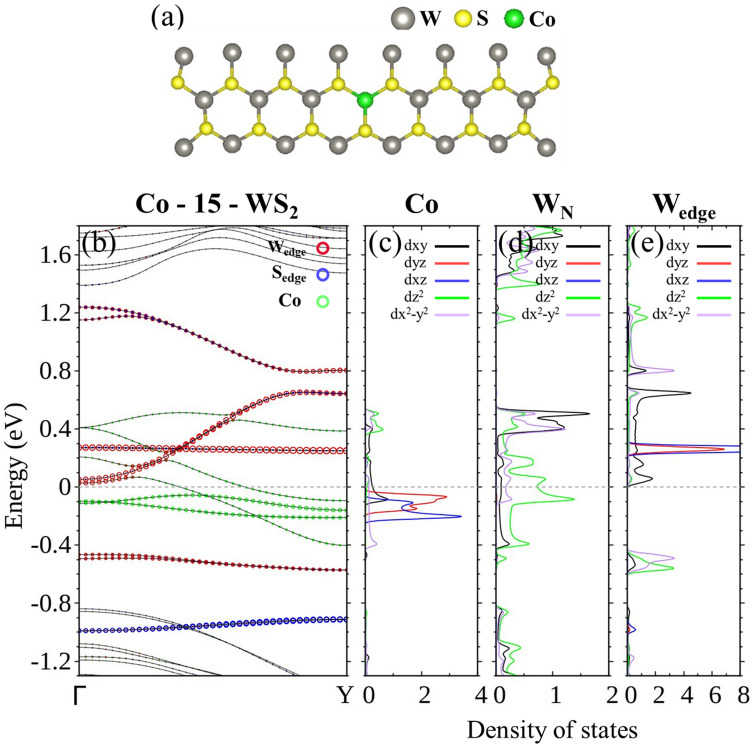
(**a**) Unit cell and (**b**) energy bands with the majority spin of Co-15-WS_2_. LDOS of the majority spin in partial waves of (**c**) Co, (**d**) W atoms closest to Co, and (**e**) W at the edges.

From partial wave analysis of the LDOS (Fig. 7), it is easy to establish that the two bands of the majority spin crossing *E*_F_ are formed by *d* orbitals of Co and the W atoms nearby, and negligibly hybridize with edge atoms in the context of conduction.

### Dopants closer to one side of 15-WS_2_

**Figure 8:**
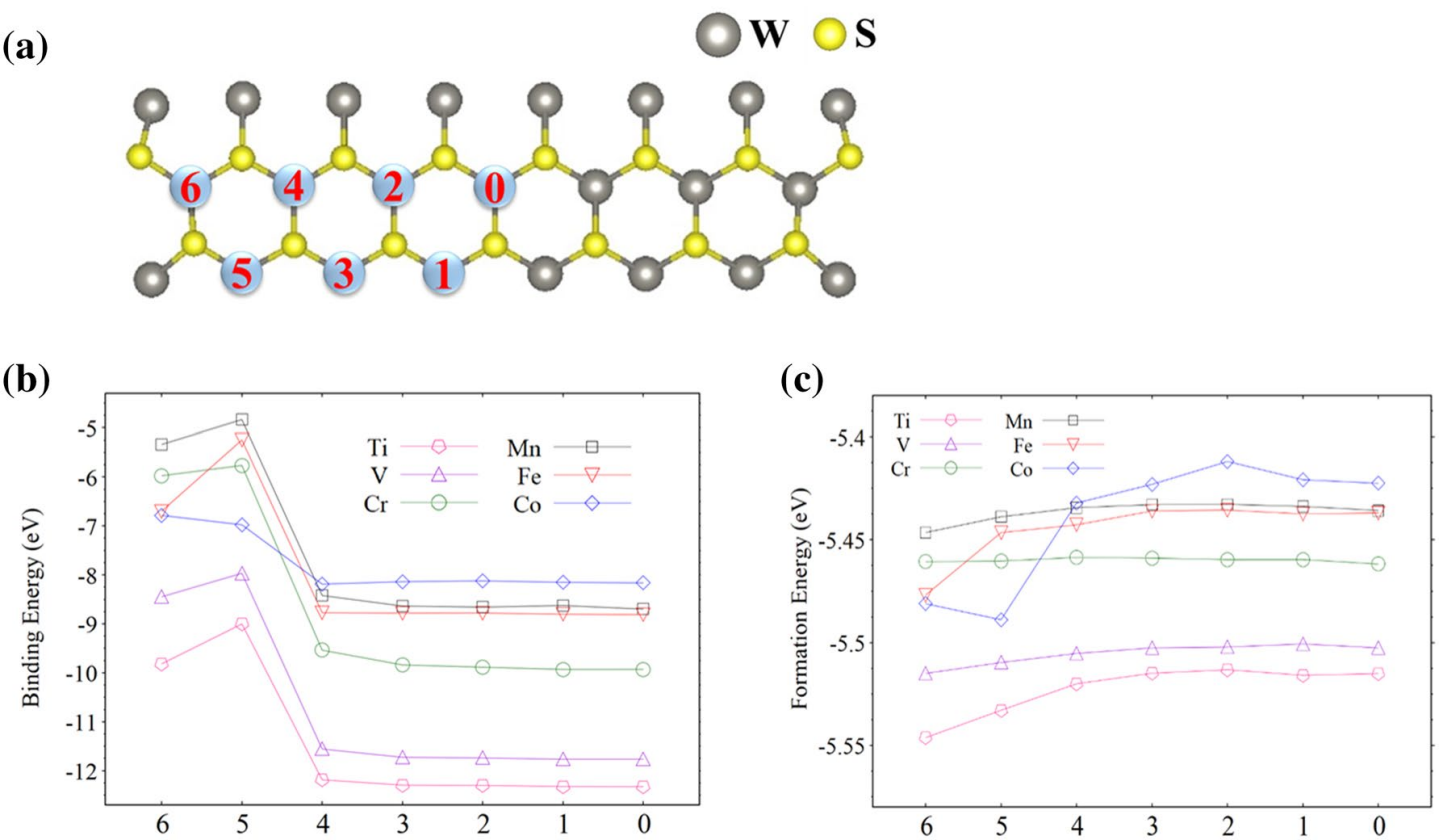
(**a**) Doping sites marked by numbers. (**b**) Binding energy for each of the six TM dopants at marked positions. (**c**) Formation energy for each of the possible configurations.

If the doped impurity atom is placed closer to one side of the nanoribbon, its wave function is expected to overlap more with those of the edge atoms on that side, and the resulting hybridization can significantly affect the electronic structure. In Fig. 8a, six sites away from the center of the 15-WS_2_ nanoribbon and numbered from 1–6 are marks for substitutional doping of the TM atoms. A larger number corresponds to a dopant that is closer to the left edge. Figure 8b plots the binding energies *E*_B_ for each dopant at the six sites and the central position (marked by 0). *E*_B_ is defined as the difference between the total energy of the doped structure *E*_*total*_(ribbon − W + dopant) and the sum of the total energies of the structure with a W vacancy *E*_*total*_(ribbon − W) and the free dopant *E*_*total*_(dopant), or *E*_B_ = *E*_*total*_(ribbon − W + dopant) − [*E*_*total*_(ribbon − W) + *E*_*total*_(dopant)]. It is clear that Ti is most tightly bound to the 15-WS_2_ nanoribbon, and each of the TM atoms is more likely to stay close to the center of the ribbon than the edges.

We also calculated average formation energy *E*_*form*_ for each of the configurations in Fig. 8b. *E*_*form*_ was derived by calculating the difference between *E*_*total*_(ribbon − W + dopant) and the sum of total energies of its constituents in isolation and then dividing the difference by the total number of atoms in the system. The results are plotted in Fig. 8c.

**Figure 9:**
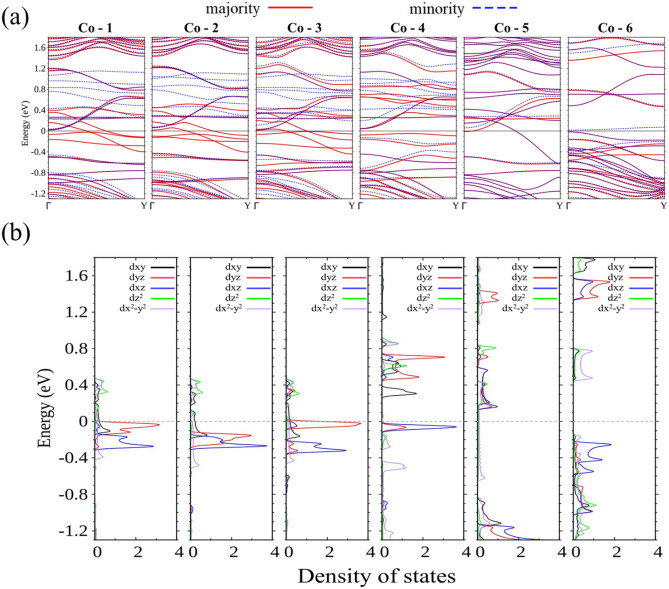
(**a**) Energy bands and (**b**) LDOS of the majority spin in partial waves for Co at sites from 1–6 (starting from the far left panel) in 15-WS_2_.

Continuing with Co-15-WS_2_, the structure remains a 100% spin polarizer with Co occupying position 1, 2, or 3. Figure 9a demonstrates that only energy bands associated with the majority spin cross *E*_F_ for the first three doping sites. If Co is placed in positions even closer to the left edge, the larger overlapping of wave functions between Co and W at the left edge significantly changes the energy bands. At positions 4 and 6, band gaps are opened and the ribbon becomes a semiconductor. Although a single band does cross *E*_F_ for the dopant at position 5, the band carries both spins and is not a spin filter. As expected, the bands responsible for conduction of the doped structure with the dopant Co at position 1, 2, 3, or 5 consist of *d* orbitals of Co (Fig. 9 b), LDOS], and its W neighbors. Part I of Supplementary Information provides a detailed illustration of the evolution of the LDOS for Co at various positions and W atoms at both edges.

**Figure 10:**
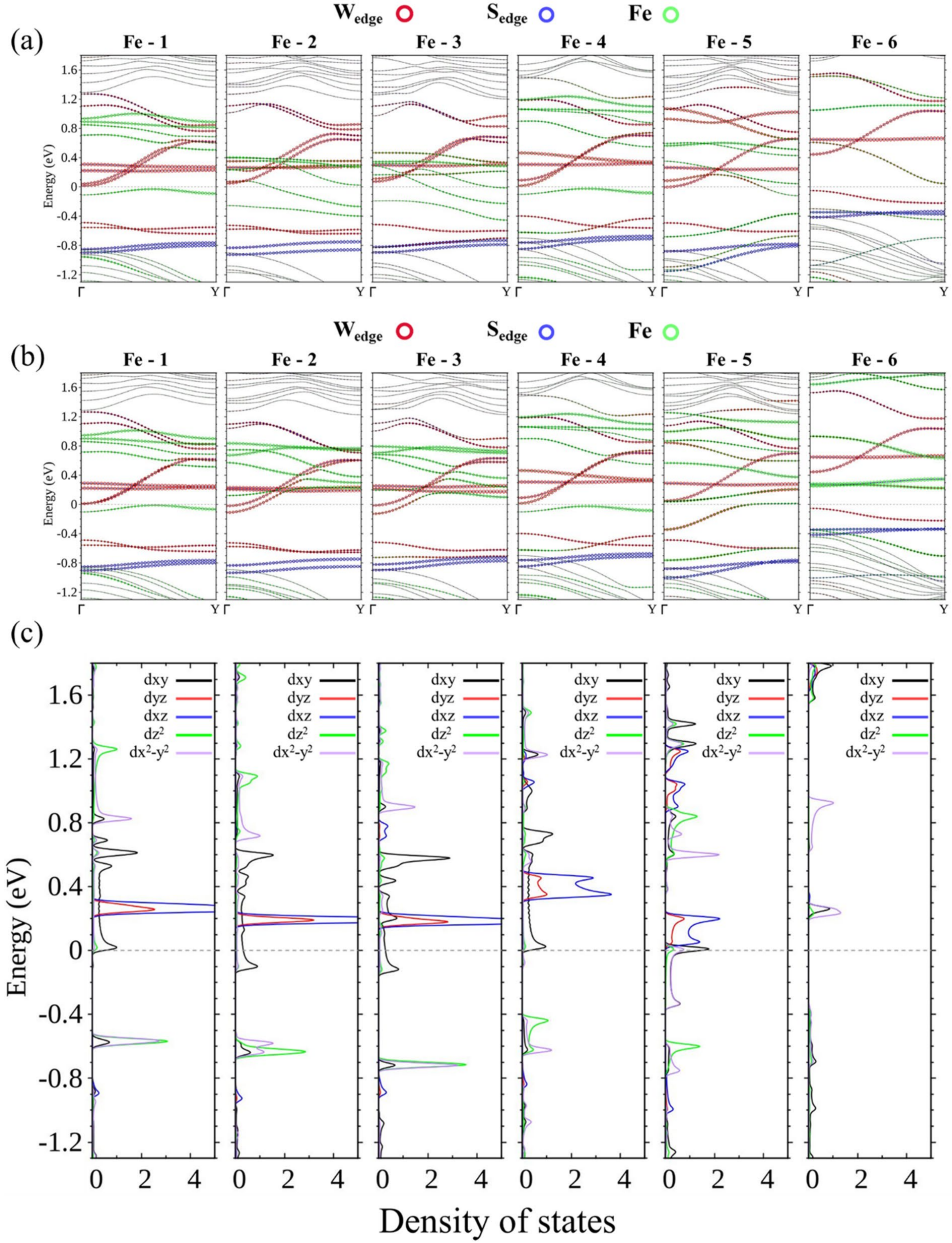
Energy bands of the (**a**) majority spin and (**b**) minority spin for Fe doping at positions from 1–6. (**c**) LDOS of the minority spin for W at the left edge with Fe occupying positions from 1–6 (from left to right).

In Fe-15-WS_2_, the Fe atom at position 1 switches the structure from a conductor to a semiconductor (Fig. 10a,b). The shortest displacement from the central position is enough to break the symmetry and induces splitting of the energy bands. Combined with the mixing of wave functions between the off-centered dopant and the W atoms at the nearer edge, a zero-gap semiconductor is created. Two-channel conduction, however, is maintained for Fe at positions 2 or 3, where transport of electrons with the majority spin is still well inside the ribbon, whereas movement of electrons with the minority spin is now limited to the left edge only, which is now closer to the dopant. Figure 10c shows the LDOS of the minority spin for W at the left edge to display the evolving distribution of *d* orbitals. The wave function of Fe at position 4, 5, or 6 is so heavily hybridized with that of W at the left edge, the LDOS curves bear almost no resemblance to the undisturbed edge states. For Fe at position 4 or 6, the structure is a semiconductor, whereas at position 5 a conductor emerges that accommodates both spins.

Similar transformations between metals and semiconductors also occur for other TM dopants placed closer to one side of the nanoribbon with the exception of Cr. Cr-15-WS_2_ is always a semiconductor regardless of the position of the Cr impurity.

### Lower concentration of dopants

It is possible to reduce the interaction between the dopant and edge atoms by widening the width of the WS_2_ nanoribbon. This is a method of reducing the dopant concentration. However, it should be cautioned that the calculation is still within the periodic framework and the system is highly ordered no matter how low the concentration is. We chose two widths designated as *N*a 20 and 25 for further study of the TM-doped system, with the dopant inevitably closer to one side of the ribbon in the former system and still occupying the central position in the latter. Ti and V-doped wider nanoribbons remain conductors through charge carriers of the dopants and their neighboring W atoms. Cr-doped ribbons are still semiconductors. Mn-doped ribbons are also semiconductors, all with small band gaps.

**Figure 11:**
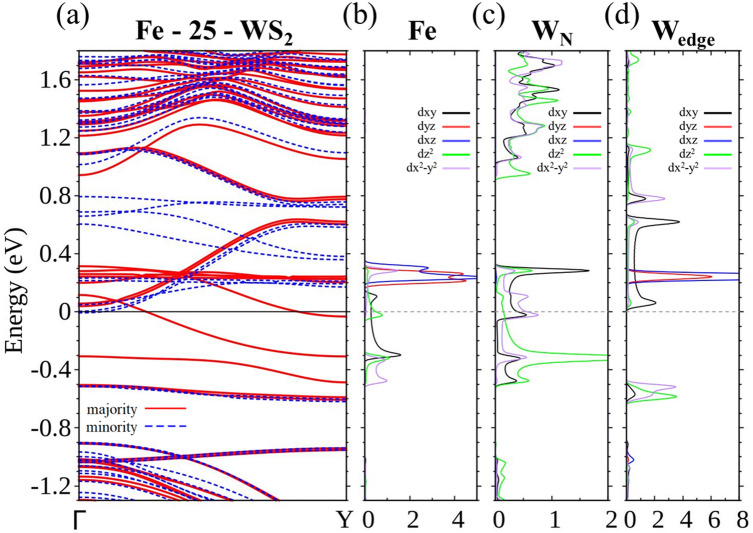
(**a**) Energy bands of centrally doped Fe-25-WS_2_. LDOS of the majority spin for (**b**) Fe, (**c**) W closest to Fe, and (**d**) W at the edges.

The most striking change occurs on Fe. As the width of the ribbon is widened, the energy bands contributed by the W atoms at the edges are raised and eventually above *E*_F_ and out of conduction. This is mainly because of less hybridization between Fe and W atoms at the edges. Fe-25-WS_2_, for example, no longer provides two-channel conduction and effectively becomes a perfect spin polarizer by only passing electrons with the majority spin. Part II of Supplementary Information shows the evolution of spin density from Fe-15-WS_2_ to Fe-25-WS_2_, and clearly indicates that the spin density at the edges of Fe-25-WS_2_ vanishes. Partial wave analysis of its energy bands (Fig. 11) also assists in establishing this picture.

**Figure 12:**
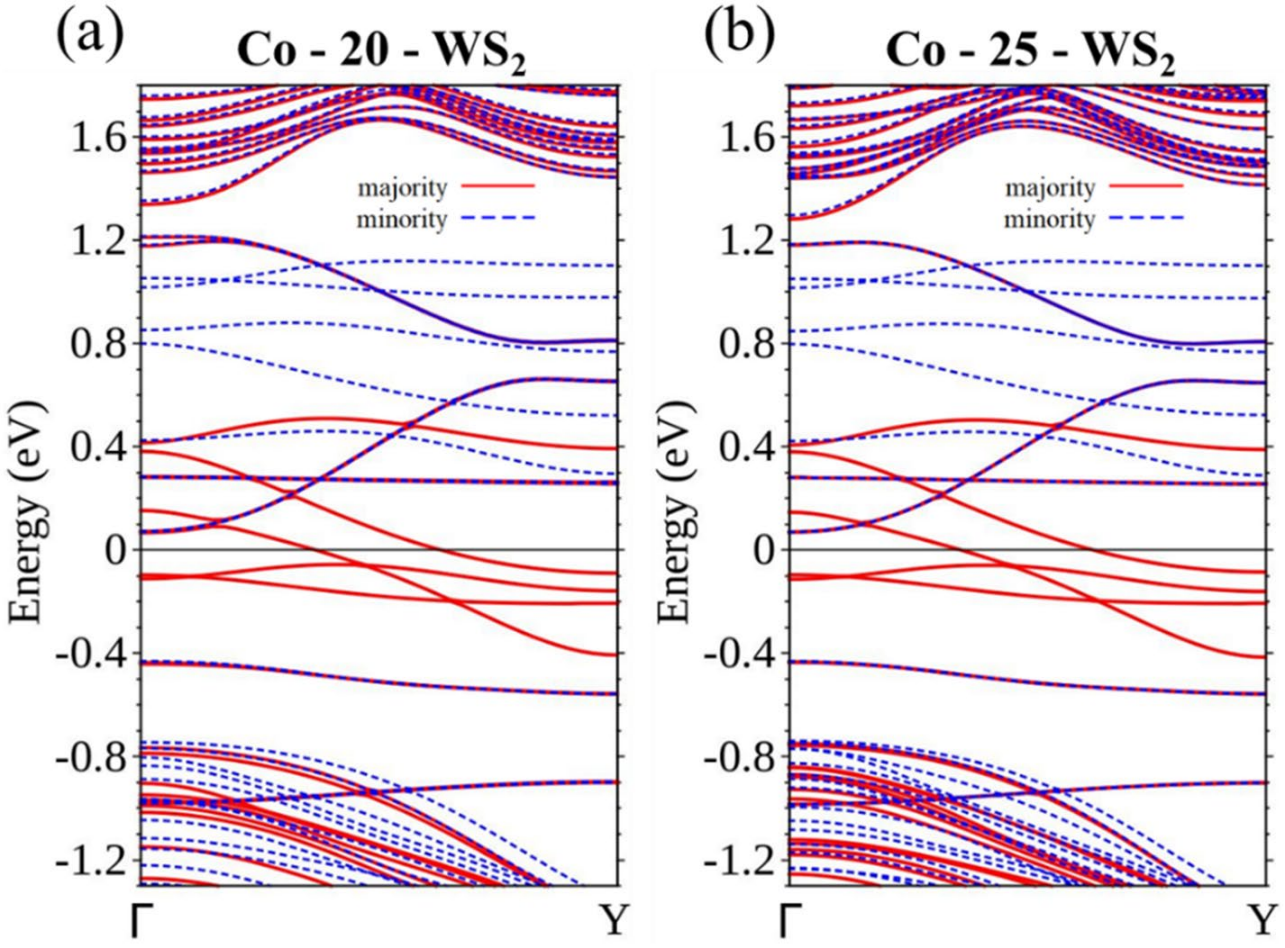
Energy bands for (**a**) Co-20-WS_2_ and (**b**) Co-25-WS_2_.

**Figure 13:**
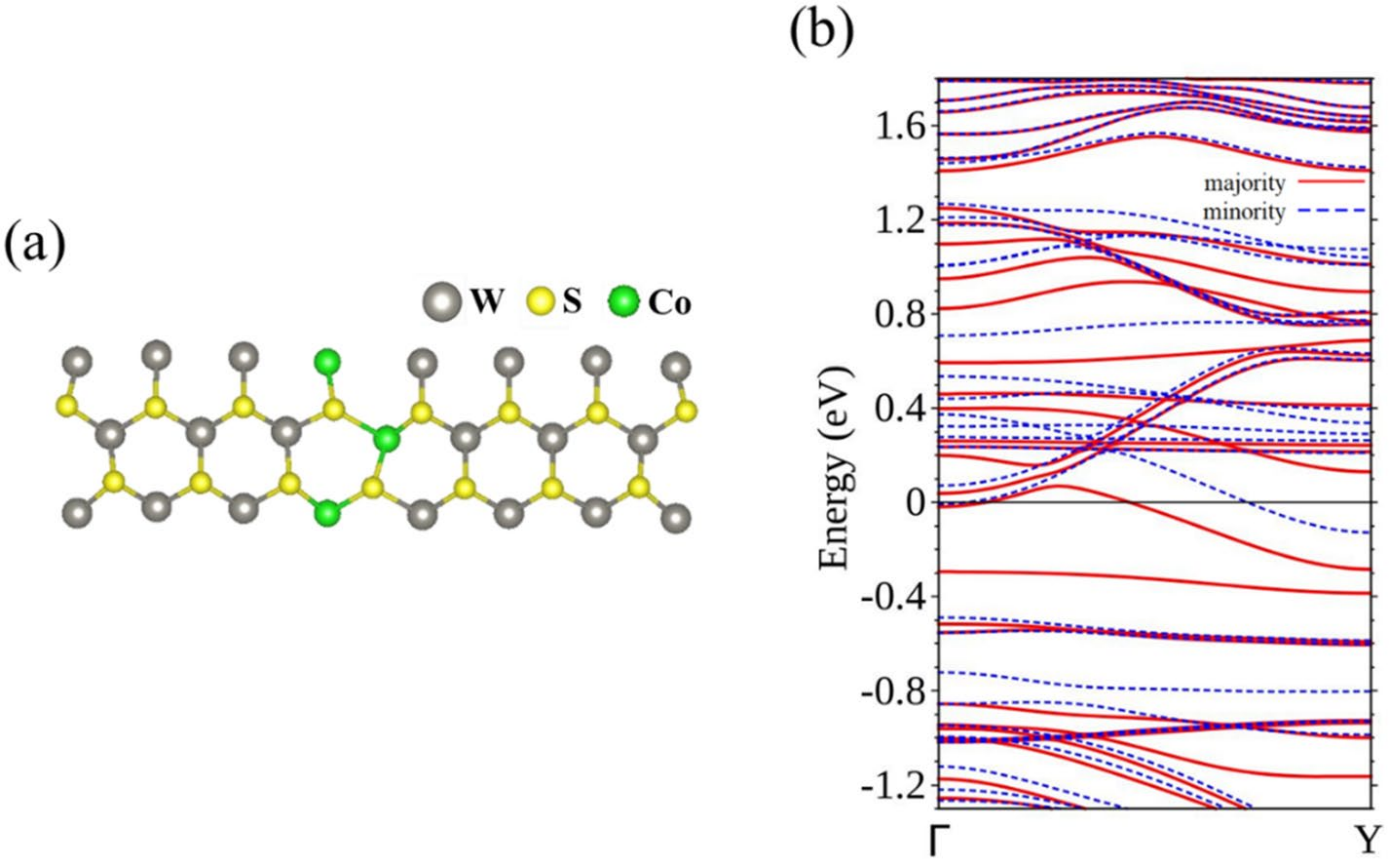
(**a**) Unit cell and (**b**) energy bands for a continuous Co chain on 15-WS_2_.

Both Co-20-WS_2_ and Co-25-WS_2_ are also spin filters with larger ribbon widths. Two bands that are associated with the majority spin crossing *E*_F_ are a feature common to both Co-doped ribbons (Fig. 12a,b), which suggests a reliable method of constructing a spin-polarized circuit through Co doping. One question arises whether a continuing Co chain (Fig. 13a, 15-WS_2_) is also a spin filter. With two bands separately belonging to different spins crossing *E*_F_ (Fig. 13b), the structure—with 2× the Co concentration and thus strengthened interaction between the dopants—becomes an ordinary conductor.

**Figure 14:**
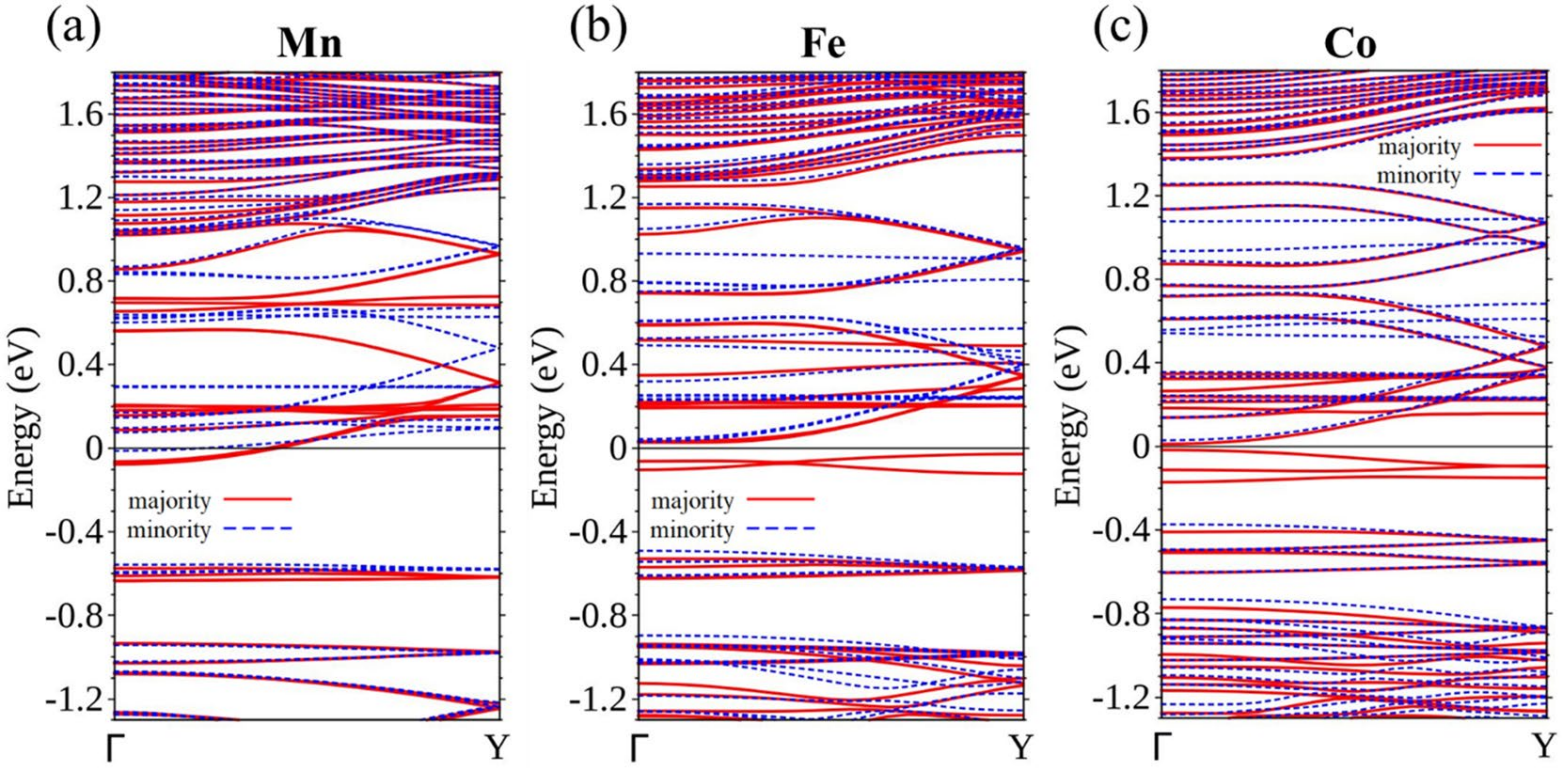
Energy bands of 15-WS_2_ for (**a**) Mn, (**b**) Fe, and (**c**) Co doping at a lower concentration of 3.33%.

Another approach for evaluating the effect of doping at a lower concentration is to increase the distance between the dopants along the ribbon length. If the distance between the dopants is doubled in the 15-WS_2_ ribbon, the concentration decreases to 3.33% and interaction between the dopants is expected to be considerably reduced. As it turns out, the lower concentration of dopants flattens their associated energy bands. Ti and V-doped structures are still conductors and the Cr-doped ribbon continues to have a band gap. Dopants of Mn, Fe, and Co behave quite differently, however, Mn-15-WS_2_ at this low concentration becomes a conductor, whereas Fe and Co each opens a small band gap (Fig. 14).

In summary, we showed that the electronic structure of an armchair WS_2_ nanoribbon can be tuned by substitutional doping of TM impurities. The dopant concentration, ribbon width, and dopant position all contribute to the doped nanoribbon’s transformation between a semiconductor and metal. Among the dopants, Fe-15-WS_2_ is capable of supporting two-channel conduction, and wider nanoribbons such as Fe-25-WS_2_ are perfect spin filters. Co-15-WS_2_ with the dopant closer to the center and corresponding wider versions also consistently support conduction of 100% spin polarization. These results will be useful for spintronics and designing nanoelectronic circuits in a broad range of applications.

## Methods

In deriving the optimal configurations of nanoribbons and energy bands, we performed spin-polarized density functional calculations using the VASP code^32,33^. Projector augmented-wave pseudopotentials and the exchange–correlation functionals of Perdew, Burke and Ernzerhof^34^ were chosen to execute the calculation. A 1 × 81 × 1 Monkhorst–Pack **k**-point mesh was used for sampling **k** points in the first Brillouin zone. We allocated a length of 20 Å in two perpendicular directions for vacuum space to eliminate artificial interaction between the supercells containing the ribbon. The cut-off energy for the expansion of the wave functions and potentials in the plane-wave basis was 400 eV.

## Supplementary information

Supplementary Information 1.
