# Supplementary material for: Electronic structures of WS2 armchair nanoribbons doped with transition metals
Source: Sci Rep. 2020 Oct 5;10:16452. doi: 10.1038/s41598-020-73602-2 (PMC7536224; doi:10.1038/s41598-020-73602-2)

**Supplementary information for the article: Electronic structures of WS_2_ armchair nanoribbons doped with transition metals**

Yan-Hong Chen^1^, Chi-Hsuan Lee^2^, Shun-Jen Cheng^1^ & Chih-Kai Yang^2^*

^1^Department of Electrophysics, National Chiao Tung University, Hsinchu, Taiwan, ROC. ^2^Graduate Institute of Applied Physics, National Chengchi University, Taipei, Taiwan, ROC.

I.


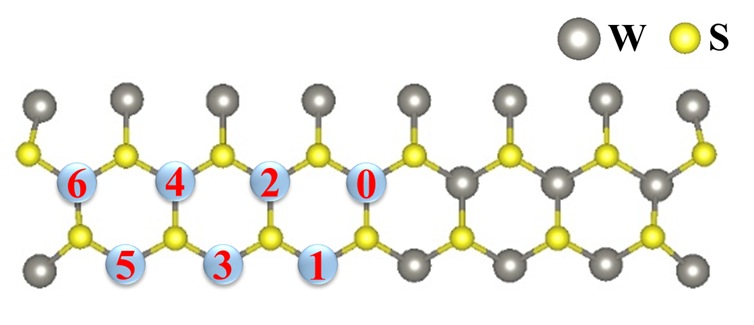


A Co atom is placed in one of the numbered positions in Co-15-WS_2_ nanoribbon.


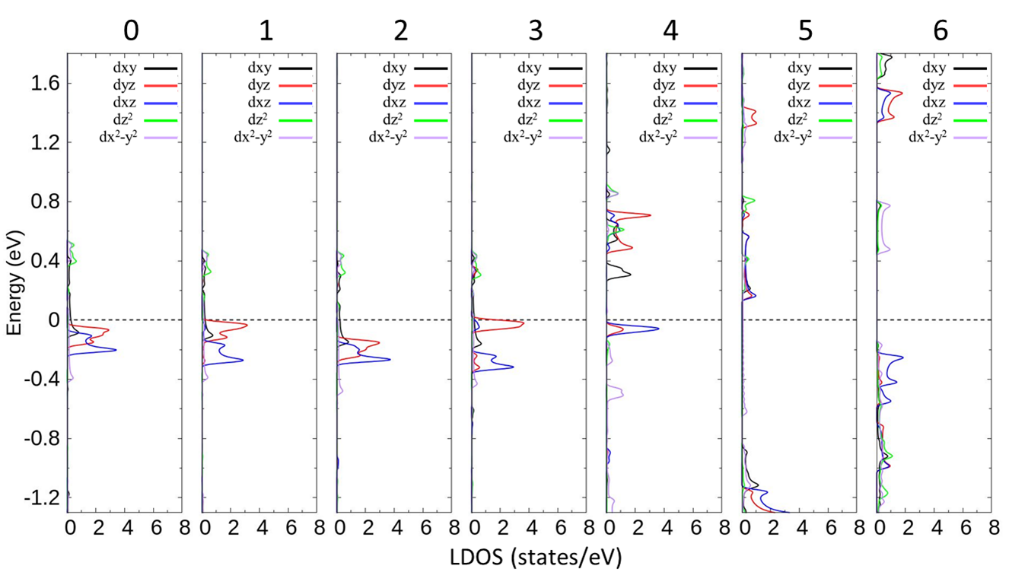


LDOS for the majority spin of Co at various positions in Co-15-WS_2_.


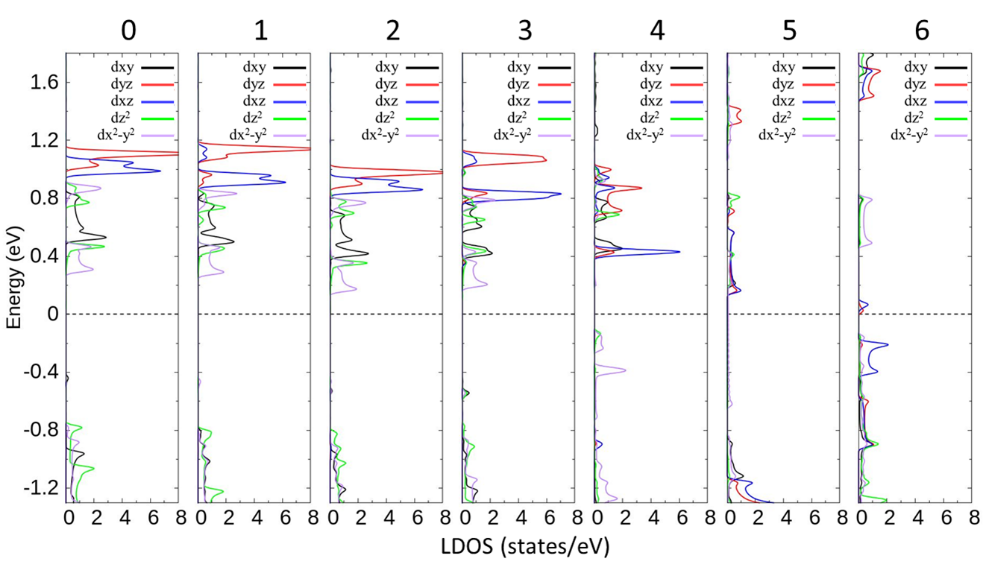


LDOS for the minority spin of Co at various positions in Co-15-WS_2_.


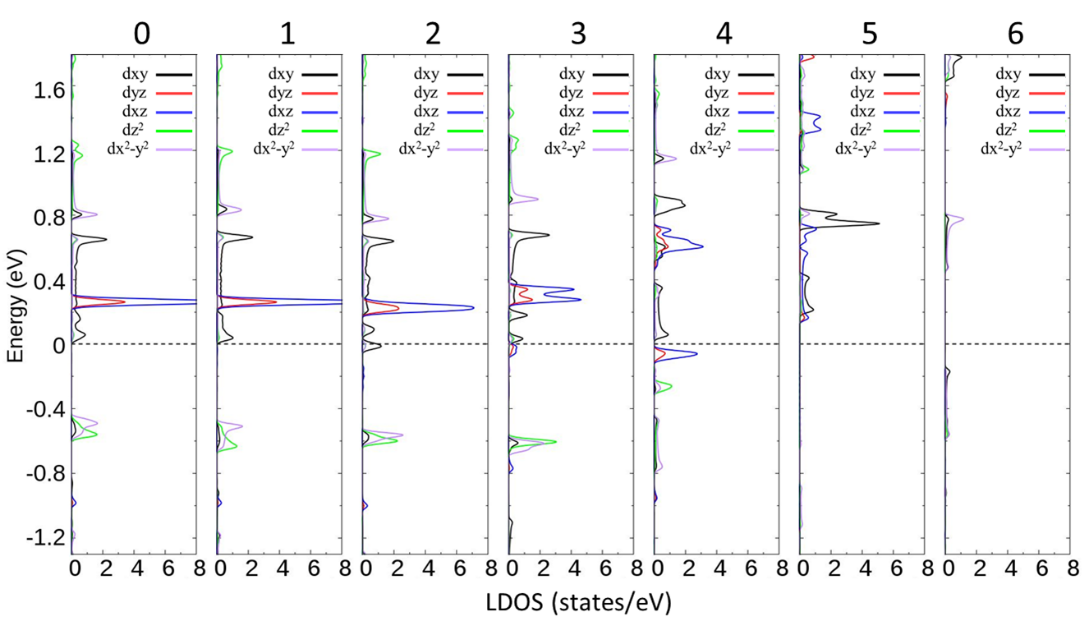


LDOS for the majority spin of W at the left edge of Co-15-WS_2_ with Co at various positions.


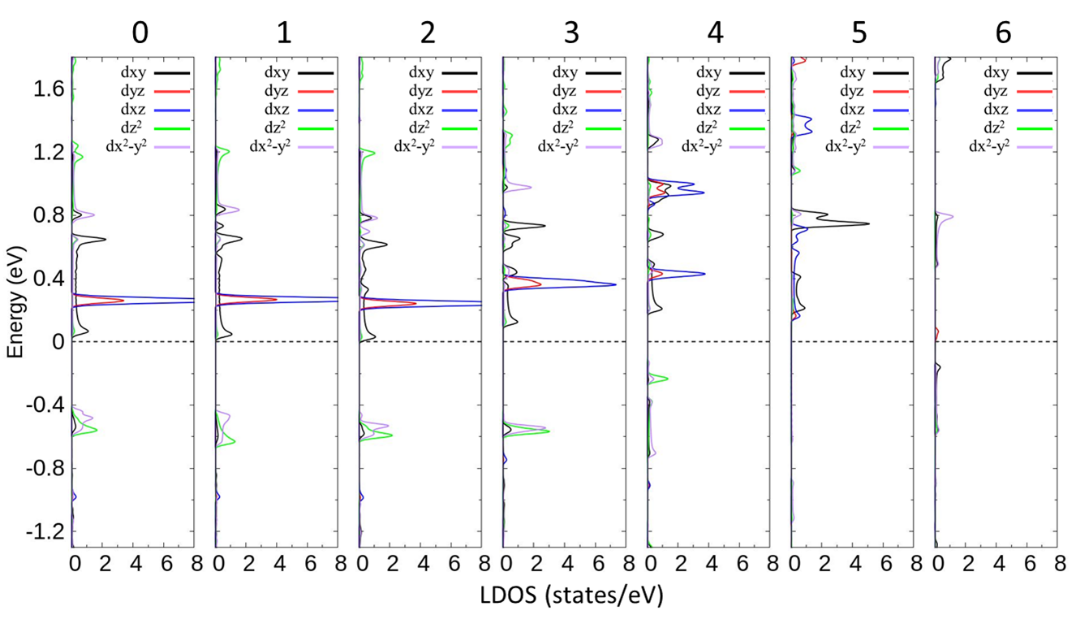


LDOS for the minority spin of W at the left edge of Co-15-WS_2_ with Co at various positions.


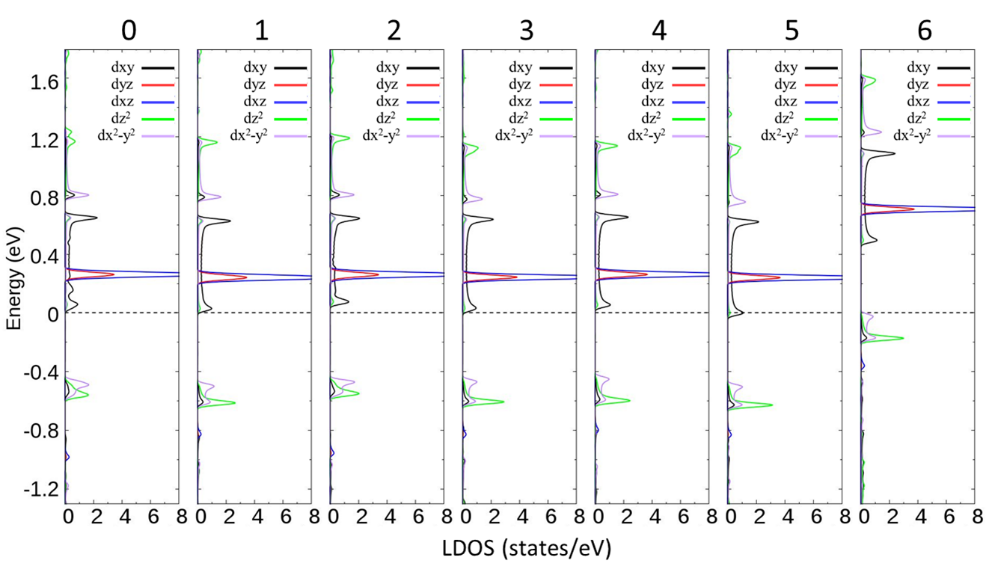


LDOS for the majority spin of W at the right edge of Co-15-WS_2_ with Co at various positions.


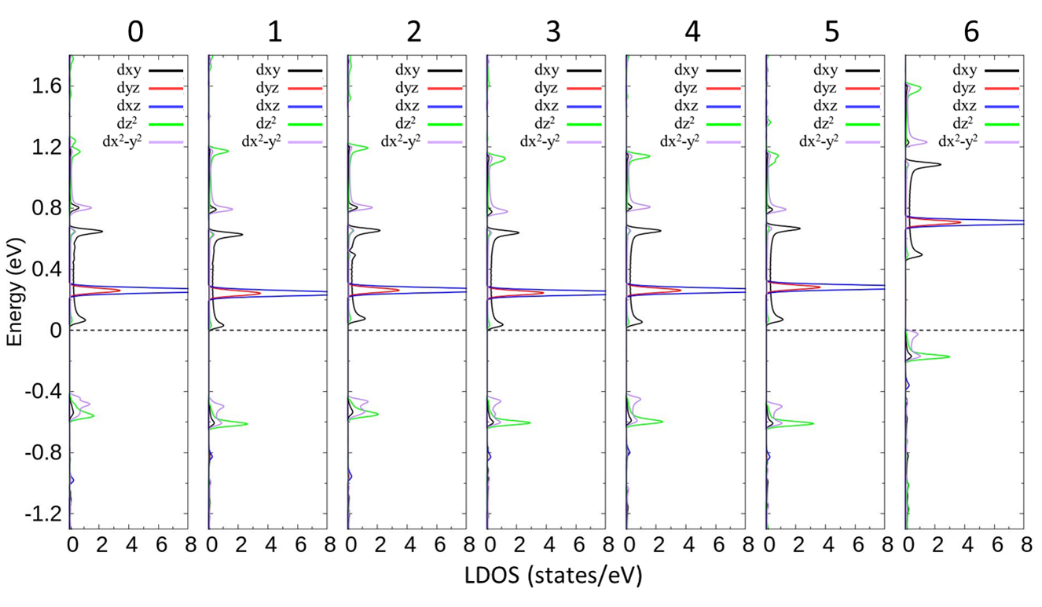


LDOS for the minority spin of W at the right edge of Co-15-WS_2_ with Co at various positions.

II. Evolution of spin density from Fe-15-WS_2_ to Fe-25-WS_2_, with red representing the majority spin and blue the minority spin.


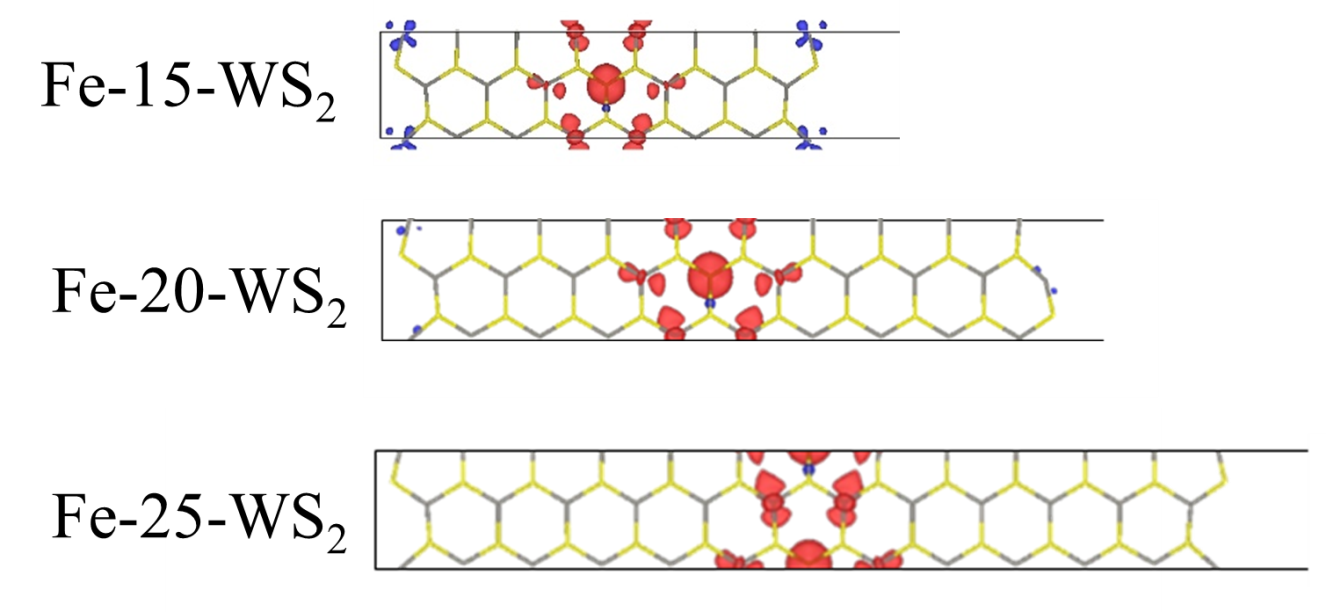

Supplement: Supplementary file 1 — Supplementary Information 1. [file 41598_2020_73602_MOESM1_ESM.docx]
